# Blindness in Childhood in Developing Countries: Time for a Reassessment?

**DOI:** 10.1371/journal.pmed.1000177

**Published:** 2009-12-08

**Authors:** Parikshit Gogate, Khumbo Kalua, Paul Courtright

**Affiliations:** 1 Lions Juhu Institute of Community Ophthalmology, Orbis-Supported Department of Pediatric Ophthalmology, H. V. Desai Eye Hospital, Pune, India; 2 Lions SightFirst Eye Hospital, College of Medicine, University of Malawi, Blantyre, Malawi; 3 Kilimanjaro Centre for Community Ophthalmology, Good Samaritan Foundation, Moshi, Tanzania

## Abstract

Paul Courtright and colleagues argue that the changing patterns of global childhood blindness suggest a need to reassess research, training, and programmatic requirements.

**Summary Points**

- Childhood blindness is a priority area for VISION 2020, a global initiative to eliminate avoidable blindness, because blind and visually impaired children have a lifetime of blindness ahead of them.
- Globally, vitamin A deficiency– and measles-related blindness in children has declined substantially although it persists in some focal settings.
- With reductions in nutritional and infectious causes of blindness, intra-uterine and genetic causes of blindness (e.g., cataract and congenital anomalies) have assumed increased importance and need tertiary care–level interventions and long-term follow-up to achieve good visual rehabilitation.
- Further research is needed to identify the underlying causes of congenital and developmental cataract and to determine the best strategies for recognition, referral, treatment, and rehabilitation.
- Changing patterns of global childhood blindness suggest a reassessment of research, training, and programmatic needs.

## Our Understanding of Childhood Blindness at the Adoption of VISION 2020 in 1999

### Magnitude of Childhood Blindness

The global initiative—VISION 2020: The Right to Sight—launched in 1999, targeted blindness in children as one of the five priority areas of disease control [1]. Selecting childhood blindness was based on the fact that blindness in childhood can lead to decades of life spent blind and because the major interventions to control childhood blindness were public health in nature (vitamin A supplementation and measles immunization) for the control of vitamin A-related corneal blindness. Childhood blindness causes significant economic burden on the family and community [2]. At the time, it was estimated that globally there were 1.4 million blind children, twice this number with low vision, and an estimated 500,000 children becoming blind each year [3]. It was anticipated that many of the incident blind would not survive because of vitamin A deficiency, so the annual cumulative annual incidence would be only about 200,000 per year [4].

### Causes of Childhood Blindness

Blindness can occur through many different pathways and due to many different pathogens or insults. Classifying the causes of childhood blindness has always been quite complicated and WHO adopted both an anatomical and aetiological classification system in an attempt to focus attention on those conditions that can be prevented, those that can be treated, and those with no prevention or treatment strategies available that would need rehabilitation [5].

Corneal scarring and phthisis bulbi due to vitamin A deficiency, measles, and infection were the commonest causes of blindness in the early 1990s in much of Asia and Africa [6]–[8]. Thus corneal blindness was listed as the primary cause of blindness in children for planning for childhood blindness [8]. In Africa, in particular, it was anticipated that corneal conditions would be responsible for approximately one-third of all childhood blindness. The correlation between vitamin A-related corneal conditions and mortality has been well documented. At the time, there was evidence to suggest that there was a positive correlation between national rates of dying under the age of 5 years (under-5 mortality rates) and the prevalence of blindness in children [9], which led to the suggestion that national under-5 mortality rates could be used as a proxy indicator of a national childhood blindness-prevalence rate for the purpose of planning [8].

### Generating Information on Childhood Blindness

Due to the rarity of blindness in children, population-based surveys to determine the prevalence of blindness require very large sample sizes and are very costly. Consequently, in the period 1980–2000, only a few population-based studies [10],[11] were carried out. A second source of information on childhood blindness are specially designed vitamin A studies in which blindness was also measured [12],[13]. Most data for making estimates of the prevalence and causes of childhood blindness, however, have come from surveys in schools for the blind or annexes [6],[7]. The limitations of using studies carried out in schools for the blind have been well recognized. Generally, children enrolled in these schools represent “past history”—that is, they developed blindness 5 or more years in the past and do not reflect current patterns of causes. Additionally, it is recognized that children enrolled in schools for the blind or annexes make up only a small proportion of the total blind in the community. Children with multiple disabilities are likely to be under-represented. Thus, generating reliable information on childhood blindness has been fraught with difficulties.

## What Have We Learned about Childhood Blindness in the Last 10 Years?

### Gradual Elimination of Vitamin A-Related Blindness

Even back in 2000 it was anticipated that “old enemies” like vitamin A-deficiency blindness would be eradicated [14]. Linking vitamin A deficiency to childhood mortality shifted vitamin A deficiency into a global public health issue rather than just a prevention of blindness issue. The number of agencies, governmental and nongovernmental, that included vitamin A supplementation and measles immunization as core programmes mushroomed. Consequently, as reported in annual UNICEF State of the World's Children reports, vitamin A-supplementation coverage has increased and blindness due to vitamin A deficiency has been virtually eliminated in many developing countries. Probably most telling was the recent report from the Lower Shire Valley of Malawi, once known as the Valley of Blindness because of the high prevalence of vitamin A-deficiency and related blindness. Compared to the previous (1983) survey [12] in which vitamin A-related corneal scars accounted for all of the children found blind, the 2007 survey [15], which used the key informant method, found that only 22% of the 37 children found blind were blind from corneal scars; 35% were blind from congenital cataract. It is possible, however, that variable study methods may account for some of the differences noted.

Regardless of the reductions in vitamin A-related corneal blindness, vitamin A deficiency still contributes to increased mortality in preschool children [16],[17]. Efforts to achieve high coverage of measles immunization and vitamin A-supplementation coverage remain just as important today as in 1999. There remain some focal areas, such as postconflict zones, refugee camps [18], and other areas [19] where vitamin A-related blindness may still occur and these areas need to be closely monitored.

### General Economic Growth and Improvements in Eye Care Infrastructure

In most developing countries general economic growth and improved health care planning have resulted in an increasing number of eye care providers, even including doctors doing private practice in rural areas. In the last 20 years Africa has seen a 4–5-fold increase in the number of ophthalmologists with increased distribution of services outside of capital cities. India has increased many fold its primary health care infrastructure in the past four decades and NGO hospitals providing high-quality low-cost eye care have been established across the country. Programmes have been put into place to reduce the use of harmful traditional eye medicines and practice, further decreasing the risk of corneal conditions [19]. There also has been easier and cheaper availability of antibiotics and better managed procurement and distribution systems. All of these health and economic infrastructural improvements have helped reduce the preventable causes of blindness.

Refractive errors are a common cause of visual impairment [20], but uncorrected refractive errors rarely cause blindness unless it is high myopia. Owing to a variety of reasons, the incidence of myopia has risen dramatically in the past two decades in east and southeastern Asia [21], however similar findings have not been detected in Africa.

### Changes in Strategies to Measure Childhood Blindness

Surveys in schools for the blind and annexes have continued [22]–[24] with the recognition, however, that many children are placed there inappropriately [25]. The shift to integrated education in some countries has led to reduced numbers of children in schools for the blind and a focus on multiple disabilities at these institutions. Thus, using surveys of schools for the blind to make estimates of causes of blindness has become less tenable.

The successful testing of “key informant” (KI) methods to find children who are blind [26] has led to the application of this method to make estimates of childhood blindness. KIs are local volunteers who live and/or work in their communities and through their vocation have a social role and are likely to know the local context, the people, and the conditions in their community. The method involves identifying and contacting the community and mapping of social networks. Local volunteers (KI) are then selected by the community and trained to find blind children in their community. An eye clinic is scheduled (2–3 weeks after training) and all of the children identified are brought to be examined by an ophthalmologist. As noted in Bangladesh, the KI method may not capture all blind children but it does provide a reasonable estimate and causes of blindness are similar to large, population-based surveys [27]. The work in Bangladesh was followed by similar activities in Ghana [28], Malawi [29], Iran [30], and Tanzania [31]. KI strategies, primarily in place to identify prevalent cases of blindness, are likely to continue to generate information in settings where there are no other practical strategies to assess childhood blindness.

### Changes in Eye Care Services for Children

While vitamin A supplementation, immunization, and improved nutrition helped decrease corneal blindness; cataracts, glaucoma, penetrating trauma, and retinoblastoma needed tertiary-level paediatric eye centres for treatment. Surgical services are expensive in terms of equipment, instrumentation, and trained human resources. The WHO recommends that there be one paediatric eye centre per 10 million population [32]. In the intervening years, there have been major investments in many parts of Asia and Africa to establish these centres. In India, in the past 10 years at least 20 new paediatric eye centres have been established, in addition to the existing four; and there are 26 centres in ten countries of sub-Saharan Africa.

With the availability of good quality surgical services has come the recognition that congenital and developmental cataract are important causes of blindness in many developing countries [33]–[35]. Furthermore, it is recognized that these centres can only be effective if they have strong links with the community and health care providers [36]. As links have been established, finding children with surgical needs has led to steep increases in numbers of surgeries being done—a typical of case of “seek and ye shall find.” At the same time, these centres have also recognized that they face significant challenges to ensure that they provide the best possible visual outcome. Delay in presentation for surgery [37] and inadequate follow-up [38],[39] remain daunting problems. The outcomes are not as spectacular as with adult cataract surgery, nonetheless the intervention substantially improves vision and a child's ability to negotiate in the world. It has been shown in developing countries that intra-ocular lenses may also be safely implanted in very young children [40],[41].

Recent studies from India put untreatable conditions such as anopthalmos and micropthalmos [22] and retinal dystrophies [42] as common causes of blindness in children, requiring strategies for rehabilitation. The etiology of congenital anomalies like anophthalmos and microphthalmos is multifactorial with a gene–environment interaction. Few studies have been able to pinpoint the exact cause [43],[44]. Even if a maternal nutritional deficiency (like folate) is implicated, interventions may be difficult to implement. Providing good quality counseling for parents and children, a high quality optometric unit (for spectacles and amblyopia management), and a low vision unit (and a larger, more diverse team), while costly, are essential to ensuring good visual outcome and proper visual rehabilitation. Older blind children continue to require good quality low vision and appropriate education opportunities. Creating links to rehabilitation programmes and educational services also has required an investment in human resources.

Retinopathy of prematurity (ROP) has been rapidly increasing in many middle-income countries, with reports from Brazil, South Africa, India, and China [45]–[48]. With better neonatal care and increased survival of more preterm babies, it seems likely that ROP blindness will become another avoidable cause unless screening programs for premature babies are implemented in the neonatology units. In order to prevent the “spread of the ROP epidemic” being experienced by Latin America and Eastern Europe, programs for prevention and treatment need to be put in place, as is occurring in many cities in India and China.

## What Next?

In least developed countries congenital and developmental cataract, retinal pathology, and congenital anomalies are gaining importance as causes of blindness in children. The relative decline in childhood factors and the corresponding increase in intra-uterine and genetic factors suggest a need for a reassessment of research, training, and programmatic priorities. Reasonable steps to be undertaken in the next 5 years include:

1. Recalculation of global estimates of prevalence, incidence, and cause of childhood blindness.
2. Investment in testing different strategies to recognize, refer, and follow up children needing surgical services.
3. Rational investment in tertiary paediatric eye care facilities along WHO recommended criteria. Coordination and collaboration between NGOs and hospitals is needed.
4. Research on the cause of congenital anomalies (e.g., anophthalmos, microphthalmos, congenital and developmental cataract, congenital glaucoma) in order to put preventive strategies in place—likely difficult and expensive steps, but nonetheless necessary to address.
5. Development of new training materials on childhood blindness, particularly for primary eye-care and secondary eye-care workers in the least developed countries.
6. Screening of premature and low birth weight babies at risk of ROP, requiring cooperation between neonatal nurses, neonatologists, paediatricians, paediatric ophthalmologists, retina specialists, and even immunization workers.

## Abbreviations

KI: key informants
NGO: nongovernmental organization
ROP: retinopathy of prematurity
